# Lane Centerline Extraction Based on Surveyed Boundaries: An Efficient Approach Using Maximal Disks

**DOI:** 10.3390/s25082571

**Published:** 2025-04-18

**Authors:** Chenhui Yin, Marco Cecotti, Daniel J. Auger, Abbas Fotouhi, Haobin Jiang

**Affiliations:** 1 School of Automotive and Traffic Engineering, Jiangsu University, Zhenjiang 212013, China; chenhui.yin@hotmail.com; 2 Advanced Vehicle Engineering Centre, Cranfield University, Cranfield MK43 0AL, UK; d.j.auger@cranfield.ac.uk (D.J.A.); a.fotouhi@cranfield.ac.uk (A.F.); 3 Automotive Engineering Research Institute, Jiangsu University, Zhenjiang 212013, China; jianghb@ujs.edu.cn

**Keywords:** maximal disk, distance transform, voronoi tessellation, centerline extraction

## Abstract

Maps of road layouts play an essential role in autonomous driving, and it is often advantageous to represent them in a compact form, using a sparse set of surveyed points of the lane boundaries. While lane centerlines are valuable references in the prediction and planning of trajectories, most centerline extraction methods only achieve satisfactory accuracy with high computational cost and limited performance in sparsely described scenarios. This paper explores the problem of centerline extraction based on a sparse set of border points, evaluating the performance of different approaches on both a self-created and a public dataset, and proposing a novel method to extract the lane centerline by searching and linking the internal maximal circles along the lane. Compared with other centerline extraction methods producing similar numbers of center points, the proposed approach is significantly more accurate: in our experiments, based on a self-created dataset of road layouts, it achieves a max deviation below 0.15 m and an overall RMSE less than 0.01 m, against the respective values of 1.7 m and 0.35 m for a popular approach based on Voronoi tessellation, and 1 m and 0.25 m for an alternative approach based on distance transform.

## 1. Introduction

Road-Layout (RL) maps are widely used in the field of autonomous driving and Advanced Driving Assistance Systems, as they provide accurate a priori knowledge of road geometry and lane markings, compensating for sensor inadequacies and uncertainties in the perception of the environment [1]. Because road features tend to follow regular geometric profiles—often straight lines—it is possible to represent these features via sparse sets of points, with negligible loss of accuracy. Sparse representations of RL maps have many advantages in real-world applications: with a lower volume of data, it is more efficient to store, label, and update a sparse representation. Given that the labelling and updating processes cannot be reliably automated and often require a few manual steps [2,3], a sparse representation allows to simplify these tedious and costly activities. Even more importantly, a smaller amount of data enable faster access and processing of the maps by path planners, visualization tools, and other relevant software components [3,4,5,6,7].

In many applications of RL maps, it is required to calculate the centerlines of individual lanes, because they provide reference trajectories and road-geometry information that are essential for the efficient implementation of trajectory planners. The centerlines are so important that they are often used in the definition of certain RL map formats [8] even though they do not usually correspond to observable features on the road. Other RL map formats, like lanelet2 [1], are defined by the road boundaries, and to convert them to formats represented by road centerlines, like OpenDrive [9], it is necessary to extract centerlines from road boundary data.

### 1.1. Related Work

So far, a variety of methods have been proposed for the extraction of the road centerlines: some are based on direct processing of road images, often taken from an aerial perspective, some are based on skeletonization of partitioned road polygons, and some are based on skeletonization of binary road maps.

When aerial images are available, road centerline extraction can be carried out using computer vision techniques, either via a two-stage process or an integrated procedure [10]. In the two-stage methods, road regions are first detected through segmentation [11,12,13,14], then road centerlines are extracted. The techniques for the second stage include morphological thinning [15,16] and learning-based algorithms [17,18]. With two-stage approaches, the accuracy and completeness of the extracted road centerlines are highly dependent on the accuracy of the road segmentation in the first step [17]. To mitigate this, integrated end-to-end methods using neural networks have been developed [17,19]. These methods are typically designed to extract high-level geometric information of road networks, and they do not provide the level of detail and accuracy required to plan trajectories useful for automated driving [20,21,22]. Compared to aerial photography, panoramic-view images captured by vehicle-mounted cameras can better describe local road scenes. Deep learning-based (DL-based) methods, like transformers, can be used to estimate centerlines directly from the camera images [23].

However, these image-based centerline estimations can suffer from the limitation of camera performance, especially in bad weather or road surface conditions [24,25]. Further, extra work is needed to capture the global connectivity of lane paths and merge local maps into a global one [26], to compensate for the limited perception range of the cameras.

Though DL-based methods have gained much popularity, the training of models relies on the ground truth of centerlines, which cannot be produced as accurately as boundary polylines by DL-based technologies [27,28] and relies heavily on manual annotation. Though some open-source datasets, like Argoverse [29] and nuScenes [30], provide centerline information, no information is provided about the methodology and the accuracy of centerlines.

Skeletonization techniques have greater potential to produce accurate centerlines for both automated driving and centerline ground truth. These approaches can benefit from the road boundary information, which is easy to obtain because boundaries are represented explicitly by visual and physical dividers, like lane markings and curbs. Prior work in this area can be categorized as contour-based and binary-map-based algorithms [31].

Contour-based algorithms rely on contour partition: the description of the target object is converted into a closed polygon composed of separated segments and scattered contour points. Several methods can then be used to extract the centerline from the resulting polygon, but the most popular solutions rely on Voronoi Tessellation (VT), i.e., the construction of a Voronoi Diagram. This technique decomposes the polygon into connected Voronoi cells, the centers of which are separate contour points. Each edge of a resulting Voronoi cell is a fragment of the perpendicular bisector of two contour points and, if the two contour points are opposite to each other, i.e., not adjacent, this fragment intersects the skeleton of the polygon [32]. Taking advantage of this property, the Voronoi edges have been successfully used as an estimate of the centerline of rivers and roads [33,34]. Delaunay Triangulation, which is considered the mathematical dual of the Voronoi Diagram [32], can also be deployed in centerline estimation. The quality of the estimated centerlines through these approximations is highly dependent on the distribution of contour points. If the contour points are scattered in an uneven distribution or at low density, the resulting centerlines tend to follow a zigzag trajectory around the real centerlines. Point intensification could be applied to the contour to increase their spatial resolution and thus produce a better approximation of the centerline, at the expense of increasing the computational cost. Furthermore, a smoothing process is usually needed to improve the smoothness of the estimated centerlines, which may cause some displacement from the real center points [34].

Among binary-map-based algorithms, thinning methods are popular. Usually, these methods iteratively remove contour pixels according to pre-defined criteria of pixel deletion, which examine the relationship between the scoped pixel and its neighborhood, until a skeleton of one-pixel width is obtained [35]. These thinning methods could be further classified into sequential and parallel algorithms, according to whether individual pixels can be processed dependently within the same iteration [36]. To improve the skeleton connectivity and erosion accuracy of thinning, hybrid thinning methods, combining different subclass approaches, are proposed [37]. At present, thinning methods are widely applied in pre-processing of pattern recognition and centerline extraction of biomedical images [38]. However, these methods usually suffer from excessive erosion of slanting patterns. And a trade-off should be made between the preservation of objective shapes and the production of one-pixel-width skeletons [39].

Instead of iteratively removing contour pixels to achieve the skeleton, distance transforms (DT) could be applied to the binary maps as a pre-processing procedure to obtain a coarse skeleton directly [40,41]. DT calculates the distance from all background pixels to their closest object contour pixels based on the given binary image. The most common metrics used in distance calculation include Euclidean, Manhattan and chessboard distance, although the Euclidean distance is often preferred because it is invariant to rotation [42].

From the distance map produced through DT, a coarse representation of the object skeleton can be created by assembling local maxima. However, the resulting set of cells is not necessarily thin enough or well-connected to represent an accurate skeleton. Skeleton patterns can be sharpened by steepest ascent methods (SAMs), as demonstrated in several solutions for ridge detection [43]. Based on this concept, ref. [44] identified so-called saddle points among local maxima in the distance map and connected them by searching for non-local maxima along the steepest uphill path, which produces connected and thin skeletons.

Another branch of binary-map-based methods that has received limited attention so far relies on the identification of the centers of the maximal inscribed circles of a polygon, defined as Maximal Disks (MDs) [45,46]. Ref. [47] calculated a signed sequential Euclidean distance map based on the outline of the skeletonization target and then run through all pixels within the outline to identify MD centers. According to the MD principle, if a selected pixel is the MD center, the distances from this pixel to its two nearest contour pixels should be equal. However, due to discretization, such pixel could not be always found. So, nine distances are calculated instead: the distance from the selected pixel to its nearest contour point, and the distance from its eight neighbors to their nearest contour points. Then a connectivity criterion based on these two distances is proposed to determine the true MD centers. Unlike the thinning method, no iteration process is needed so MD is more efficient. To avoid generating redundant branches from the skeleton, ref. [48] introduced a contour partition process, based on the discrete curve evolution, before exploring MDs. Through partition, the integrated contour is divided into several individual segments according to the pattern topology. Then MDs are generated in the way that their touching points with the contour are associated to different segments, whose loci are the main skeleton of the pattern with no spurs. Although skeleton extraction using MDs can be efficient, the extraction accuracy might be reduced due to the phenomenon known as ligatures [45]. In the deployment of the MD principle, multiple MDs might touch a single contour pixel. Ideally, an infinite number of MDs could be generated, rotating around this shared pixel and constrained by polygon contours, thereby forming a curved skeleton. The section, consisting of this curved skeleton, is referred to as a ligature. Since MDs are explored within a binary map, the curved skeleton is approximated by several discretized pixels, which results in some loss of accuracy.

In general, a more accurate skeleton can be obtained as the resolution increases in the distance map; however, the implementation is usually computationally prohibitive, as confirmed by the results presented in Section 3. While DL-based methods have become increasingly popular, they face several limitations, particularly in scalability, computational cost, interpretability, and geometric accuracy. During training, DL methods heavily rely on diverse samples to generalize effectively and are sensitive to data imbalance. In inference, they often require significant computational resources, such as GPUs, to operate efficiently. Additionally, DL methods function as “black boxes”, which limits their interpretability and does not guarantee precise geometric accuracy.

### 1.2. Motivations and Contributions

The analysis of prior work shows that the methods for the extraction of high-accuracy centerlines from a sparse representation of the road boundaries tend to rely on spatial resolution enhancements, which further increase the computation and storage burden. The aim of this paper is to propose a solution to calculate the minimal set of points that accurately describes the centerline of road lanes in the sparsely defined scenarios, without changing the representation through re-sampling, interpolation, discretization or filtering. This approach enables a compact description of RL maps, efficient usage of computation and memory resources, and fast labelling of aerial views.

The novel contributions of this paper are:

1. A novel approach for the extraction of the road centerline is proposed, based on MD. Instead of re-sampling the space, MDs are formed directly based on the constraining elements, either segments or their extreme points taken from the road boundaries.
2. In addition to the identification of the relevant MDs, a criterion is proposed to link the centers of the MDs into a connected centerline.
3. To relieve the computation burden, a segment pairing method to assemble suitable constraining elements is also presented.
4. To improve the centerline calculation accuracy, ligatures are identified and additional circles are supplemented to reduce errors in the ligatures.
5. To overcome the limitations of the available public datasets, and to control the effects of road geometry and sample density, a custom dataset has been generated based on relevant parameters.
6. Performances of the proposed approach and two popular skeletonization methods, based on VT and DT, are compared in sparse and dense scenarios from both a self-created and a public dataset of road layouts.

This work is significant to the robotics, mapping, and transportation systems communities because:

1. By producing a minimal set of centerline points, the proposed method reduces the size of RL maps, is memory-efficient and, while its computational cost is comparable to other skeletonization methods, the reduced amount of data can save computational resources down the processing pipeline, typically in trajectory planning applications.
2. The proposed method can be used to convert RL maps from border-based to centerline-based formats, retaining only the significant data points. This simplifies the manual maintenance of the produced RL maps. Additionally, the generated sparse representation of the RL can aid in road network analysis and ease the load for path planning applications.
3. This paper provides valuable data on the accuracy, computational cost, and memory usage of the three methods mentioned above (MD, VT, and DT), allowing researchers to select the best approach when performing centerline extraction in dense and sparse scenarios.
4. This method can automatically produce accurate centerlines that can be used as ground truth to train DL-based solutions.

In the rest of this paper, Section 2 presents the centerline extraction method based on MDs, as well as the proposed implementation of the solutions based on VT and DT. Experiments and results are reported and discussed in Section 3, followed by the conclusions drawn in Section 4.

## 2. Methodology

A centerline extraction method based on the MD principle is proposed in this section. The process consists of two steps: calculating center points based on MD and then linking center points to form a centerline. The details of the method are elaborated on in the following paragraph.

To provide a comprehensive analysis, two alternative methods are explored based on the VT and DT techniques. In centerline extraction applications, the VT method is particularly advantageous for its high time efficiency, especially when dealing with low-resolution border points, and it ensures accurate estimations in scenarios of high spatial density. On the other hand, DT-based solutions are well applied when objects are represented in polygonal forms, such as lanes in our case. They offer extremely accurate estimation of centerlines at high resolutions, making them suitable for providing ground truth in comparative analysis within this research. Both methods have demonstrated their efficacy in a variety of centerline extraction tasks across different domains, including medical imaging, road morphology and biometric identification [49,50,51,52]. Their proven effectiveness in centerline extraction justifies their selection for comparison with our proposed MD-based method.

### 2.1. The Extraction of Lane Center Points Based on MD

**Figure 1:**
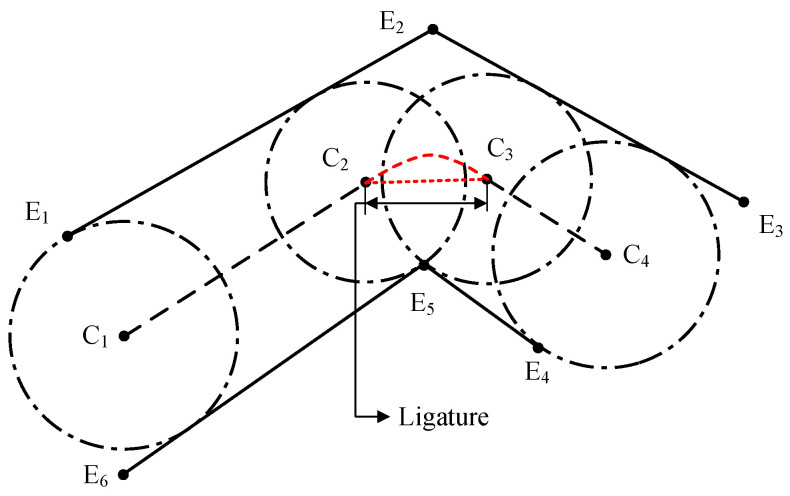
Illustration of a combination of two-lane pieces. $E_{i}$ are extreme points of the line segments. $C_{i}$ are circle centers of the maximum circles. The red straight dotted line denotes the ligature. And the curved dashed red lines are estimated centerlines in the ligature.

In a road map defined by sparse border points, a road lane can be partitioned into a set of connected lane pieces, each of which consists of 2 opposite line segments and their extreme points. A lane example is illustrated in Figure 1. According to the MD principle [45], the lane centerline extraction consists of finding and linking the centers of the maximal inscribed circles. In Figure 1, an example of a maximal circle is the circle centered at $C_{1}$, which passes through one extreme point and is tangent to one of the opposite line segments. $C_{1}$ is equidistant to both of the opposite lane border segments that it touches, and thus is on the bisector of the segment pair. So is $C_{2}$, and all centers of the maximal circles touching these two segments lie on the same bisector segment defined by $C_{1}$ and $C_{2}$, which is also the centerline. Taking advantage of this property, it is possible to reduce the amount of data used to represent the centerlines of a simple lane to only two points, without loss of accuracy. Unfortunately, this approach cannot be applied directly in the presence of sharp bends and complex lane structures, so the aim of this paper is to identify the minimum number of MDs that can be used to represent the lane centerlines in any sparsely described scenario and to increase the approximation accuracy in the bends.

When considering sharp bends that are sparsely described, it is important to notice that the centerline might rotate around one of the extreme points, so an infinite number of MDs is needed to accurately represent this line. This is recognized as a ligature and is depicted as a red dashed line in Figure 1. The approximation of the curve in the ligature by a straight segment creates a shortcut path and results in some loss of accuracy, causing the convex curve to flatten. In this work, the term “primary MD” will be used to identify MDs that are uniquely constrained by a group of constraining elements, or that represent the start or the end of a ligature, while the term “secondary MD” will be used for the infinite number of MDs along the ligature.

**Figure 2:**
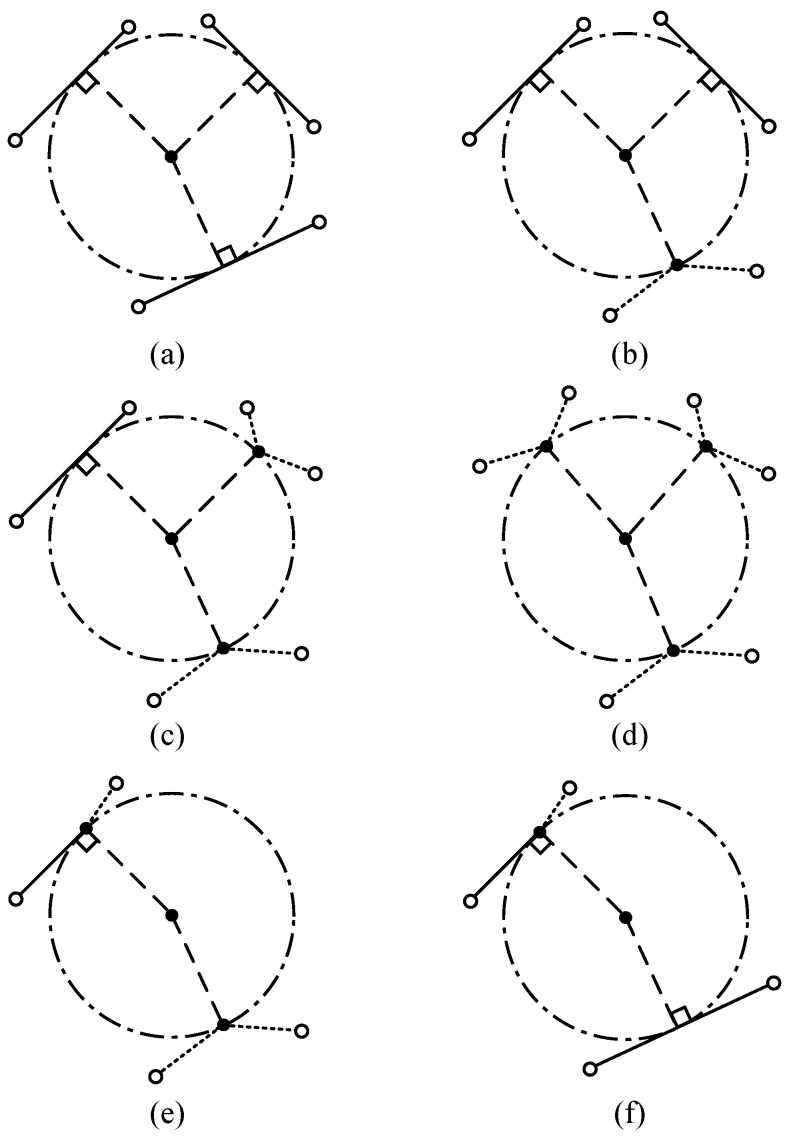
Illustration of maximal inscribed circle types. Two types of constraints: the circle being tangent to a segment or passing by a point. In (**a**), the circle is constrained by 3 segments. In (**b**), the circle is constrained by 2 segments and 1 extreme point. In (**c**), the circle is constrained by 1 segment and 2 extreme points. In (**d**), the circle is constrained by 3 extreme points. In (**e**) the circle is constrained by 1 segment and 2 extreme points, with one of the two points lying on the segment. In (**f**), the circle is constrained by 2 segments and 1 extreme point, which lies on one of the two segments.

To mitigate this problem and improve the accuracy of the centerline, several cases of inscribed circles are explored. An illustration of how inscribed circles can be related to surrounding lane segments is given in Figure 2.

According to the number of segments and points constraining the MD, four basic cases can be identified, shown as Figure 2a–d. In these basic cases, the segments and the points constraining the MD are not related, but there are also two special cases, shown as Figure 2e,f, where one of the constraining points lies on one of the segments.

Of all the six cases, Figure 2f is the most common one in road scenarios, as it is characteristic of the beginning of a new lane piece with a slight bend. The other cases are related to special configurations of the road boundaries, including sharp bends, and road bottlenecks. Since the coordinates of all border points are known, the formulation of the line segments could be given in the standard form:

(1) Ax+By+C=0,

where *A*, *B* and *C* are the parameters of the standard line equation of the line element.

The distances from the circle center to the tangent line segment and the passed extreme point could be calculated as:

(2) $$dist_{c2p}={\left(x_{c}-x_{p}\right)}^{2}+{\left(y_{c}-y_{p}\right)}^{2},$$

(3) $$dist_{c2l}=\frac{{\left(Ax_{c}+By_{c}+C\right)}^{2}}{A^{2}+B^{2}},$$

where $dist_{c2p}$ and $dist_{c2l}$ are distances from the circle centers to the point and line constraint elements separately, $x_{p}$ and $y_{p}$ are the coordinates of the point element, $x_{c}$ and $y_{c}$ are circle center coordinates.

Taking case (b) as an example, the constraints of the point and line segments could be expressed by:

(4) $$\begin{array}{c} \left\{\begin{array}{cc} {\left(x_{c}-x_{p}\right)}^{2}+{\left(y_{c}-y_{p}\right)}^{2} & =\frac{{\left(A_{1}x_{c}+B_{1}y_{c}+C_{1}\right)}^{2}}{A_{1}^{2}+B_{1}^{2}},\\ {\left(x_{c}-x_{p}\right)}^{2}+{\left(y_{c}-y_{p}\right)}^{2} & =\frac{{\left(A_{2}x_{c}+B_{2}y_{c}+C_{2}\right)}^{2}}{A_{2}^{2}+B_{2}^{2}} \end{array}\right. \end{array}$$

which denotes that the circle center has equivalent distances to one point and two line segments.

More generally, Equation (4) could be written in the form of:

(5) $$\begin{array}{c} \left\{\begin{array}{cc} A_{1}x_{c}^{2}+B_{1}y_{c}^{2}+2C_{1}x_{c}y_{c}+2D_{1}x_{c}+2E_{1}y_{c}+F_{1} & =0,\\ A_{2}x_{c}^{2}+B_{2}y_{c}^{2}+2C_{2}x_{c}y_{c}+2D_{2}x_{c}+2E_{2}y_{c}+F_{2} & =0 \end{array}\right. \end{array}$$

where $A_{c}$, $B_{c}$, $C_{c}$, $D_{c}$, $E_{c}$ and $F_{c}$ are known:

(6) $$\begin{array}{c} \left\{\begin{array}{cc} A_{c1/2} & =e_{1/2}A_{1/2}^{2}-1,\\ B_{c1/2} & =e_{1/2}B_{1/2}^{2}-1,\\ C_{c1/2} & =e_{1/2}A_{1/2}B_{1/2},\\ D_{c1/2} & =e_{1/2}A_{1/2}C_{1/2}+x_{p},\\ E_{c1/2} & =e_{1/2}B_{1/2}C_{1/2}+y_{p},\\ F_{c1/2} & =e_{1/2}C_{1/2}^{2}-x_{p}^{2}-y_{p}^{2}, \end{array}\right. \end{array}$$

And in the Equation (6):

(7) $$\begin{array}{c} \left\{\begin{array}{cc} e_{1} & =\frac{1}{A_{1}^{2}+B_{1}^{2}},\\ e_{2} & =\frac{1}{A_{2}^{2}+B_{2}^{2}}. \end{array}\right. \end{array}$$

In a similar way, all six cases can be represented starting from Equations (2) and (3), and the circle center could be found by solving this non-homogeneous binary quadratic system.

### 2.2. Segment Pairing

The previous section explains that at least two separate border segments or some of their extreme points are required to constrain an MD, so it is possible to identify the MDs by searching for suitable sets of segment pairs. It is important to notice that at least two of the three border constraints (either segments or extreme points) have to be associated with separate boundaries, i.e., either the right or the left border, otherwise they are constraining a cove, which is irrelevant for centerline calculations. It would seem reasonable to assume that segment pairs are somehow “overlapping” with each other, meaning that they are vaguely in front of each other. However, as Figure 2 shows, this relationship is not always straightforward. To overcome this problem and avoid a computationally expensive search across the entire map, this paper proposes to search for neighboring segments.

**Figure 3:**
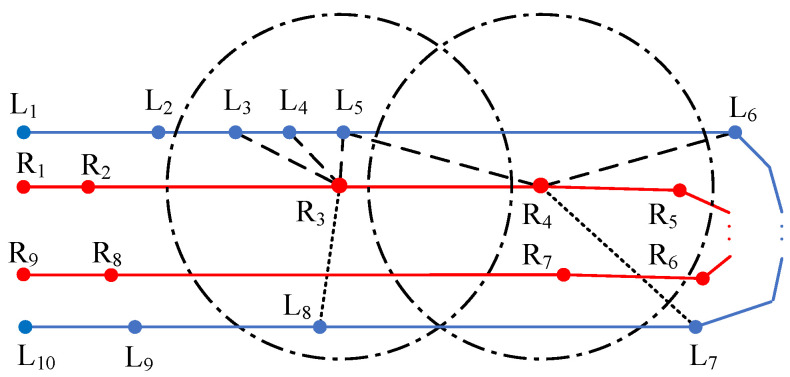
An illustration of a range search and a nearest–neighbor search, based on point $R_{3}$ and point $R_{4}$ separately. Blue line segments belong to the left lane border while red ones belong to the right lane border. In the range search, $R_{3}$ should be matched with $L_{3}$, $L_{4}$ and $L_{5}$, but not $L_{8}$. In the nearest neighbor search, $R_{4}$ should be matched with $L_{5}$, and $L_{6}$, but not $L_{7}$.

The search starts with the selection of an extreme point of a segment. A circle centered at this point is formed with a fixed customized radius and all points within the circle, on the opposite border, will be considered as potential constraining points for an MD, or extreme points of a constraining segment. This can be seen as a range search procedure. The result of a range search is shown as all points inside the circle centered at $R_{3}$ in Figure 3.

Some of these identified points are unsuitable for matching, because belonging to a section of the border that is unreachable by an MD. This situation is common in a U-type lane, as shown in Figure 3. An in-lane MD could not be found under the constraints of $R_{3}$ and $L_{8}$ or their relevant line segments. So $L_{8}$ should not be matched with the selected extreme point $R_{3}$. To remove these unwanted points like $L_{8}$, accumulated distances along the border are considered as the point filtering criteria. The circle points inside the search range are first grouped by their indexes, in which the point indexes are numerically continuous and increase from the lane start to the end in sequence. For example, $R_{3}$-relevant points will be divided into two groups, one containing $L_{3}$, $L_{4}$ and $L_{5}$ and the other containing $L_{8}$ only. Then, the group of points with smaller indexes will be chosen if the accumulated distance of the selected extreme point to the start is smaller than its distance to the end; otherwise, the group of points with bigger indexes will retain. As Figure 3 shows, the extreme point $R_{3}$ is closer to the start point $R_{1}$ and the group containing $L_{8}$, having a larger index, is discarded.

With the matched points, segment pairs could be established by constructing segments according to the point indexes.

Due to the sparse distribution of the points, no opposite point may be found within the given circle in the range search. But a point pair does exist. An example can be seen in Figure 3 that no points are found in the circle centered at $R_{4}$ but an MD might exist with constraints of points $R_{4}$ and $L_{6}$ and their relevant segments. To avoid this omission, a nearest-neighbor search is performed after the range search to supplement its limitation on the search scope. All points on both borders are stored in the structure of a 2-dimensional tree [53] for easy and quick point query. The number of nearest neighbors is set to 3 to ensure correct and efficient point pairing. And the same point filtering method as used in range search in this section is used to remove points failing to constrain an in-lane MD.

### 2.3. Circle Centers Filtering

**Figure 4:**
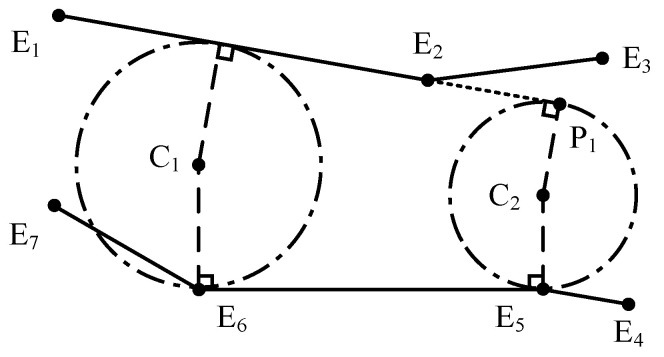
Example of MDs that need to be filtered out. The circle $C_{1}$ is crossed by border segment $E_{6}E_{7}$, thus is beyond the lane region. The contact point of the circle $C_{1}$ with segment $E_{1}E_{2}$ is on the segment extension, which has produced a circle beyond the scope of segment constraint.

Through segment matching and circle center extraction, all possible circles are found. But some circles interfere with the lane borders (like $C_{1}$ in Figure 4), or the contact points are not on the border segments but their extension (like $C_{2}$ in Figure 4). These circles have either exceeded the domain of the lane or overstepped the constraint of their related segment pairs. Thus, those circles that are not completely inside the lane polygon, or are weakly constrained by their related segment pairs, are deleted.

### 2.4. Connectivity of Circle Centers

The previous section describes how to select a minimal set of primary MDs, whose centers are lying on the centerline. The centerline, however, is defined as an ordered sequence of points, so it is necessary to organize and connect the obtained circle centers.

As illustrated in Figure 2, the MDs touch a segment through contact points, which are either tangent points on the segment or its extreme points. The position of these contact points can be used to sort the MDs to form a centerline.

**Figure 5:**
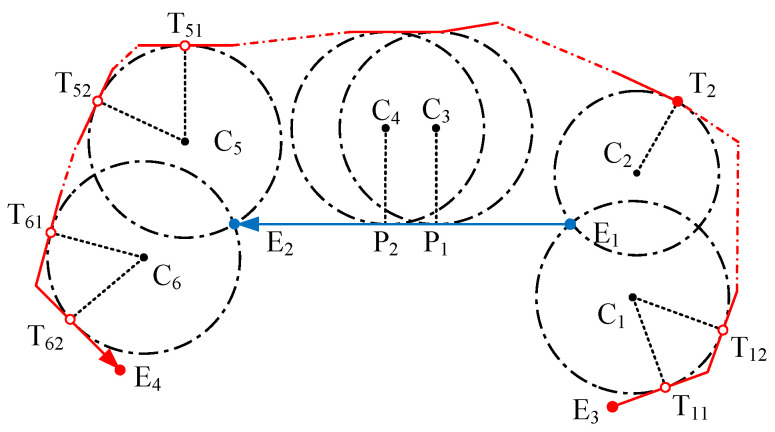
The left lane border, in blue, and the right lane border, in red, are oriented according to the direction of the arrows. The associated MDs are shown together with their contact points.

At the beginning of the connecting process, the MDs are sorted according to the contact points on a chosen border, based on the border-along distance between the contact point and the start point of the border. For example, in Figure 5, $C_{3}$ comes earlier than $C_{4}$ because its contact point $P_{1}$ is closer to $E_{1}$, the start point of the segment, than $P_{2}$.

In case of a tie (typically, two MDs passing by the same extreme point), the MDs are sorted according to the contact points on the opposite border, based on the border-along distances to the start point of the new border.

It is not unusual for some MDs to touch one of the borders with two projection points, like $C_{1}$, $C_{5}$ and $C_{6}$ in Figure 5. In these cases, the projection point that is closer to the border start point participates in the sorting. For example, in Figure 5, the start point at the right border (in red) is $E_{3}$. Consequently, $T_{11}$ is chosen over $T_{12}$ in the sorting of $C_{1}$. As a result, $C_{1}$ comes earlier than $C_{2}$ because the contact point $T_{11}$ has a shorter border-along distance than $T_{2}$.

### 2.5. Compensation for Ligature

As is shown in Figure 1, a ligature starts and ends up with two primary MDs $\left(C_{2}\;\mathrm{and}\;C_{3}\right)$ connected by a common extreme point $\left(E_{5}\right)$. Between these two primary MDs, additional secondary MDs can be created with two constraint elements: the same extreme point $\left(E_{5}\right)$, and a tangent line segment (for example, the line segment $E_{1}E_{2}$).

While these two constraints apply to an infinite number of secondary MDs, the range of the touching points on the line segment is defined—they will lie between the two touching points of the two primary MDs. By sampling a new touching point within this range, a secondary MD can be produced with the constraints of this touching point, the tangent line segment and the extreme point. This is the same as Case (e) shown in Figure 2.

For simplicity, the line segment is represented as its start point $E_{1}$ and a normalized direction vector $\vec{L_{1}}$. Any point *P* on the line segment can be represented with its distance to the segment start point along the line direction:

(8) $$P=E_{1}+k\vec{L_{1}},$$

If *P* is the projection of a secondary MD with circle centre *C*, its distance can be calculated as the dot product of the direction vector of the line segment with the vector from the start point to the MD center:

(9) $$k=\vec{L_{1}}\cdot \left(\vec{C}-\vec{E_{1}}\right),$$

Denote the two primary MD centers’ as $C_{1}$ and $C_{2}$, the resulting projection-to-start distances are $k_{1}$ and $k_{2}$ separately. The projection points of secondary MDs between these two primary MDs have a range of $\left(k_{1},k_{2}\right)$. With a selected *k* in this range, a new secondary MD can then be calculated. While an infinite number of values can be sampled, this work proposes, as a trade-off between efficiency and accuracy, to take 5 evenly spaced samples in this range, and the resulting secondary MDs are used to compensate for the ligature.

### 2.6. The Pipeline of the Proposed Method

The step-by-step pipeline to get a centerline based on the proposed MD method is shown in Algorithm 1 below.
**Algorithm 1:** Centerline Calculation based on MD**Require:** Surveyed points of both road lane borders: $pts_{\mathrm{left}},pts_{\mathrm{right}}$**Ensure:** Calculated centerline 1:Build a 2-dimensional tree for the points of each border: $KDT_{\mathrm{left}},KDT_{\mathrm{right}}$ 2:**Perform segment pairing:** 3:**for** idx_border=1:2 **do** 4:    **for** $\mathrm{idx}\_\mathrm{pt}=1:{\mathrm{ptNum}}_{\mathrm{left}/\mathrm{right}}$ **do** 5:        Extract a query point queryPt from $pts_{\mathrm{left}/\mathrm{right}}$ 6:        Perform range searching based on queryPt and $KDT_{\mathrm{left}/\mathrm{right}}$ to form point pairs 7:        Do point-pair filtering based on Section 2.2 8:        **if** point pair is not found **then** 9:           Perform nearest-neighbor search10:           Do point-pair filtering based on Section 2.211:        **end if**12:    **end for**13:    Build segment pairs $segPairs_{\mathrm{left}/\mathrm{right}}$ by the point pairs based on Section 2.2 and create a list of them for each segment14:**end for**15:**Explore MDs of six cases:**16:**for** idx_border = 1:2 **do**17:    **for** $\mathrm{idx}\_\mathrm{seg}=1:{\mathrm{ptNum}}_{\mathrm{left}/\mathrm{right}}-1$ **do**18:        Select one segment and extract relevant segment pairs from $segPairs_{\mathrm{left}/\mathrm{right}}$19:        Select constraint elements (points and segments) to calculate MDs of six cases20:        Do circle-center filtering based on Section 2.321:        Store the circle centers $circleCentres_{\mathrm{idxBorder}}$22:    **end for**23:**end for**24:**Connect the circle centers:**25:Extract either left or right border $pts_{\mathrm{left}/\mathrm{right}}$26:**for** $\mathrm{idx}\_\mathrm{seg}=1:{\mathrm{ptNum}}_{\mathrm{left}/\mathrm{right}}-1$ **do**27:    Select one segment from the border28:    Extract circles touching the segment29:    Sort the circles according to Section 2.430:**end for**31:**Compensate for ligature:**32:Identify ligatures by primary MDs sharing the same constraint point33:**for** idx_lgt=1:lgtNum **do**34:    Calculate the projection-to-start range35:    Sample new projection points36:    Calculate the secondary MDs in the ligature37:**end for**

### 2.7. The Extraction of Lane Centerlines Based on VT

The procedure for centerline estimation based on VT is inspired by Golly et al. [34] but with some changes: the dead-end branches are filtered by the shortest path search between the start and end nodes instead of by counting Voronoi edge connections, and no further smoothing is performed on the Voronoi edges. Specifically, the steps below are proposed:

1. Define a closed lane polygon by linking scattered lane border points sequentially.
2. Perform Delaunay Triangulation inside the lane polygon [54].
3. Operate VT [32].
4. Filter and prune the Voronoi edges.
5. Connect Voronoi edges into one consistent line.

**Figure 6:**
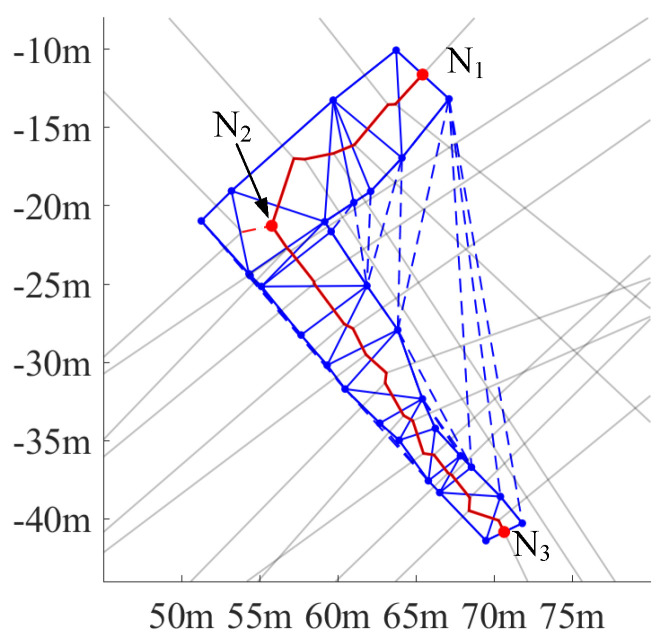
An illustration of the centerline (solid red segments) estimated by the VT. The grey lines are Voronoi edges perpendicular to border segments. The solid blue segments are the resulting edges from inside–lane Delaunay triangulation, while dashed blue segments are edges of Delaunay triangles outside the lane polygon. $N_{1}$ and $N_{3}$ are the start and end nodes of the centerline separately, and $N_{2}$ is a branch node. The branch node has three connected edges, one of which is a residual spur (the red dashed line).

Lane polygons are usually nonconvex and, as a result, Delaunay triangulation will be performed both inside the lane polygon and in its concavity, as shown by the dashed blue triangles Figure 6. These triangles should be removed because they do not belong to the lane region. The result of this operation is shown in Figure 6 by the solid blue lines.

VT is performed after triangulation. The resulting Voronoi edges are represented by the grey lines and the red lines in Figure 6. The grey lines are edges perpendicular to border segments, the lane entrance, or the lane exit. And, because they do not contribute to the construction of the centerline, they need to be filtered out. After this filtering, there might still exist some spurs, like the dashed red line in Figure 6. To remove these spurs and achieve a well-connected centerline, the start and end nodes of the centerline are firstly identified by searching for the intersection points of the Voronoi edges and the predefined entrance and exit segments (like the nodes $N_{1}$ and $N_{3}$ in Figure 6). Then, a unidirectional path search is performed to find the path connecting the start and end nodes through the remaining Voronoi edges. In this way, edges belonging to the centerline will be connected in sequence, and the spurs will be removed. The output centerline is shown as the red line in Figure 6.

### 2.8. The Extraction of Lane Centerlines Based on DT

The proposed centerline extraction method, based on DT, can be organized into four main steps:

1. Project lane boundaries on a binary grid of appropriate resolution.
2. Perform 8-neighbor Euclidean Distance Transform inside the lane polygon [42].
3. Operate ridge detection with 8-neighbor Discrete Laplace Transform [43].
4. Find and connect centerline points by Dijkstra’s Searching [55].

In the first step, the lane boundaries are projected on a binary grid by assigning a value of 1 to the cells intersecting with them, and a value of 0 to the non-intersecting cells. The entrance and exit of the lane polygon are not projected to the binary grid to avoid meaningless values of distance that cause a bifurcation of the centerline, as shown in [56].

**Figure 7:**
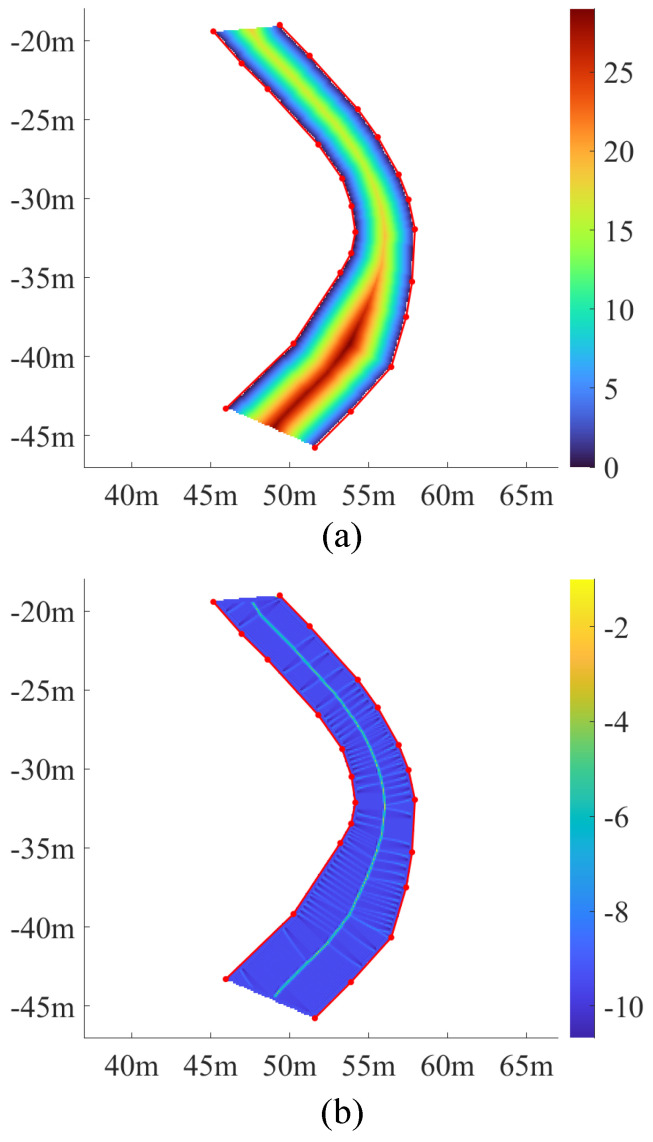
Visualization of the results of the intermediate steps of centerline extraction based on DT. (**a**) The distance map. (**b**) The ridge map.

A distance map is then calculated from the binary map, by propagating the distance from the boundary to the neighboring cells, across the whole map [42]. The metric of 8-neighbor Euclidean Distance is used to provide a smooth variation of the distance value. To reduce the computational load, the DT is restricted inside the lane polygon with a created in-lane discriminant matrix. Furthermore, the distance between two orthogonally adjacent cells is set to 1 instead of the resolution of the grid map. This means that the intensity of the ridge in the ridge detection will not change with the resolution. The resulting distance map is shown in Figure 7a. The colored area is the area of interest, and the different colors represent the distance values derived from DT.

A process is then applied to sharpen the several-pixel-width central domain: the Discrete Laplace Transform (DLT) is used to obtain a ridge map by evaluating the second-order difference between the cell of interest and its neighbors in the distance map [43]. The 8-neighborhood discrete Laplacian of a cell is used in this research.

Figure 7b illustrates the results of the DLT. The central area has shrunk to a line, exhibiting a several-pixel width. At the same time, some secondary ridges have emerged in the transverse direction, due to the discrete nature of the computation.

To find the final one-pixel-width centerline points and to connect them in order, Dijkstra’s search is deployed. Selecting and linking the center points is done in conjunction with the Dijkstra’s search, by tracking the local minima of the ridge map, corresponding to the location of the ridge. Since Dijkstra’s search is typically designed to operate in the positive domain and the ridge map is mostly negative, a new map is calculated by adding the absolute value of the global minimum to the value of each cell in the ridge map. The cost function is set to find the minimal accumulated values of the resulting map.

## 3. Experiments and Results

The centerline extraction results based on Maximal Disks (MD), Distance Transform (DT) and Voronoi Tessellation (VT), are compared in the following section in terms of computational efficiency and performance accuracy.

### 3.1. Data Description

The performances of three centerline calculation algorithms are evaluated based on both self-created and public road datasets. The self-created dataset allows for a methodical analysis of the general performance of three methods, by controlling the effect of construction parameters for the road boundaries. The public road dataset enables further investigations into the performance of the MD method and its most competitive counterpart, based on the VT, on complex intersections of roads.

**Figure 8:**
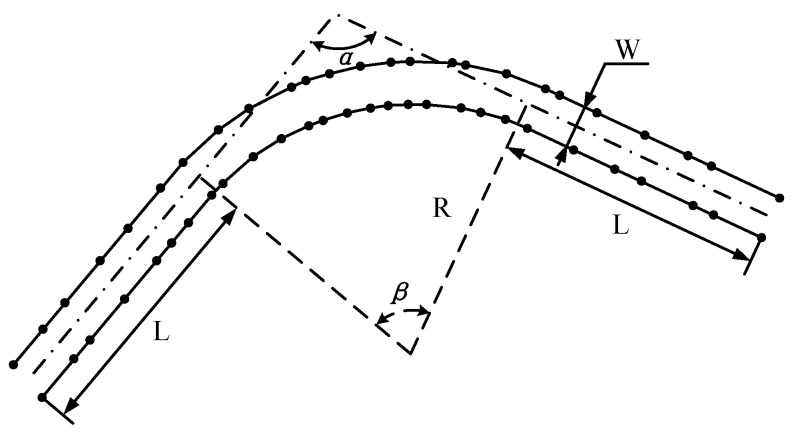
An example of a constructed road. α is the intersection angle of two arms. β is the central angle of the curve. *R* is the radius of the curve. *L* is the length of the road arms. *W* is the width of the road. The black points are sampled points on the road borders.

In the self-created dataset, the road structure and geometric parameters are designed according to road construction policies shown in [57]. Each road is composed of two straight arms and a curve with a tangent-circle-tangent connection. This geometric topology mimics the general road structures in real worlds, like streets and highways, and covers both the straight through and turning maneuvers. An example of a constructed road is shown in Figure 8. A special case of two arms intersecting with each other directly without curved transition is also considered, which could be seen as lane merging scenarios or very sharp corners.

In detail, the overall length of each road is set to 150 m and the two arms have equal length. Each road structure is characterized by a set of arm intersection angle α, curve radius *R* and road width *W*. Different combinations of these three features introduce different challenges for the three methods. For example, a corner with a small curve radius will cause MD to produce ligatures that generate large centerline estimation errors. Increasing road width will downgrade the performance of VT when the spatial resolution of the contour points stays the same.

For simplicity, these parameters are constrained by a common range, following the instructions in [57], and sampled by a constant step. The intersection angle α varies between 75 and 105 degrees with a step of 15 degrees. The radius *R* varies according to the minimum road curvature requirements and values of 8, 16, 50 and Inf are used, representing scenarios from narrow turnings to wide curved lanes. An Inf is used in the case that two arms intersect directly with no transition curve, which mimics a very sharp corner. The road width *W* ranges from 1.2 to 3.6 m, typical widths from a narrow footpath to a wide driveway. A total of 36 complete roads are created by the combination of the three road structure parameters.

The road border points are sampled randomly by the Latin Hypercube Sampling (LHS) method [58]. LHS is selected because compared to random sampling, it is more likely to preserve the original lane geometric features. Generally, the point density is low on the straights and it is high on the corners, to better retain the shape of curves.

**Figure 9:**
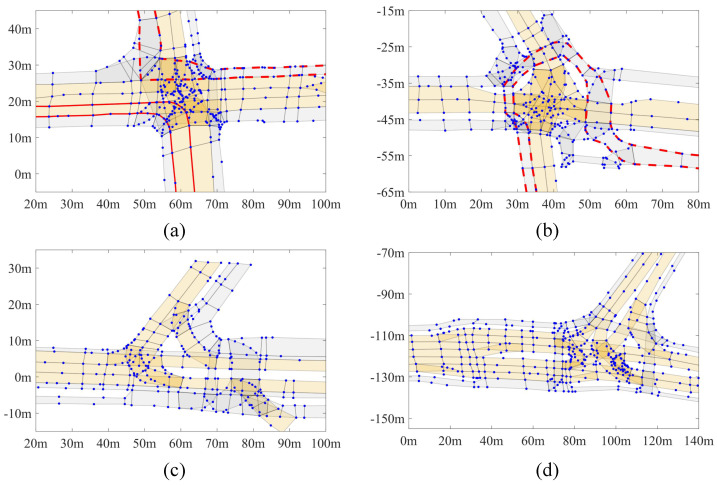
The four scenarios in the inD are illustrated in (**a**–**d**). Sparse points are marked in blue. Lanelets are represented by polygons. Vehicle driving lanes and non-driving lanes (like pedestrian paths) are marked in light yellow and grey separately. Examples of a complete pedestrian path and a driving lane are bounded by solid and dashed red lines separately.

The selected public dataset is the ‘inD Dataset’ [59]. Four scenarios are included in the inD and the corresponding map elements are represented by sparse points. An illustration of the four scenarios is shown in Figure 9. For clarity, the four distinct scenarios are named Sce1, 2, 3 and 4 in the following performance experiments. To have a general overview of the intersection geometries, readers can refer to [59] for more details.

Geometric information like lane coordinates is presented in the Lanelet2 format [1] and a lane is defined as a series of connected lanelets, which are the basic elements in the map and are modelled by polylines. To extract a complete lane for centerline calculation, initial lanelets are identified first, which have no predecessors. Then these initial lanelets are extended by their successors until final lanelets are reached, which have no further successors, to finish the road lane construction. By this method, 27, 34, 20 and 22 lanes are extracted from Sce1–4 respectively, including both driving and non-driving lanes.

**Figure 10:**
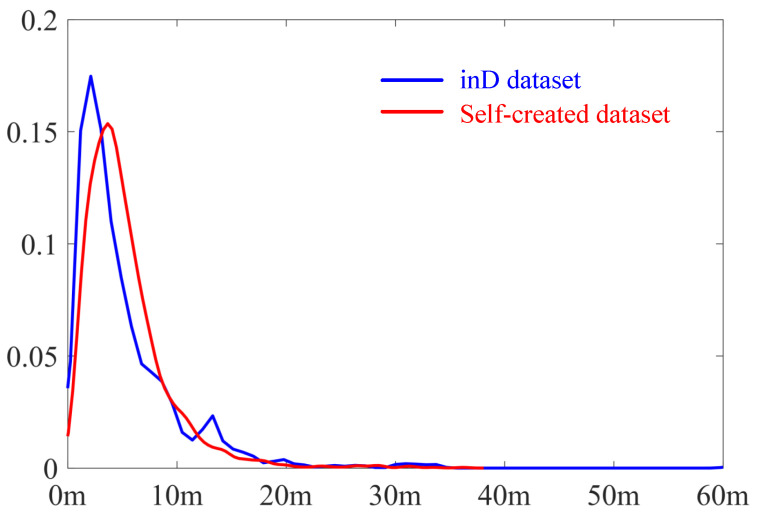
Illustration of KDE results of boundary segment length. The bule curve represents the inD dataset while the red one represents the self-created dataset.

In this study, both the self-created dataset and the inD dataset are presented in a sparse format. The sparsity is defined by the average segment length (avrgSegLgth) of the surveyed lane boundaries, which quantifies the mean distance between consecutive boundary points. Table 1 provides detailed statistics on these lane boundaries. For simplicity, the self-created dataset is abbreviated as SCD in the table. In addition to avrgSegLgth, other measurements, the number of lanes extracted (numLanes), the total length of all lanes (accuLgth), as well as the maximum (maxSegLgth) and minimum segment lengths (minSegLgth) in each scenario, are provided to offer a comprehensive overview of sparsity. Additionally, the distribution of boundary segment length is measured by kernel density estimation and is illustrated in Figure 10.

From Figure 10, both datasets exhibit a wide distribution of boundary segment lengths and share similarity. In Table 1, it is notable that the maxSegLgth can reach up to 88.85 m and the avrgSegLgth measures several meters. This is sparse, especially when compared to the high-density requirements of certain applications. For example, dynamic trajectory planning typically demands point spacings of less than 1 m [60], while density-sensitive methods like VT require resolution finer than the minimum road width for accurate centerline extraction (usually within several decimeters) [34]. This point is further validated by the tests discussed in Section 3.2. To provide an overview of road widths across scenarios, Table 2 summarizes key statistics. This includes the average width (avrgWidth), maximum width (maxWidth), minimum width (minWidth), and the standard deviation of road lanes in the scenario. The road width is estimated using the MD radius, which is an inherent output of the proposed method.

### 3.2. Experimental Results and Comparisons

All methods are implemented and simulated using MATLAB software (R2020b), on a computer with an AMD R7-4800H processor and 16 GB RAM. It is important to note that the implementation of the MD approach is based entirely on custom code, which offers plenty of opportunities for optimization, while the VT and the DT take advantage of very mature libraries from Mathworks (delaunayTriangulation, voronoi, bwdist, conv2, shortestPath).

First, experiments are run on the self-created dataset. In terms of computational efficiency, the total execution times (ET) of centerline extraction of different methods are recorded. For a fair comparison of individual approaches, the time required by data loading and storing processes is ignored. Each script is run multiple times and the median of the ETs is selected. The resulting ETs are illustrated in Table 3.

**Figure 11:**
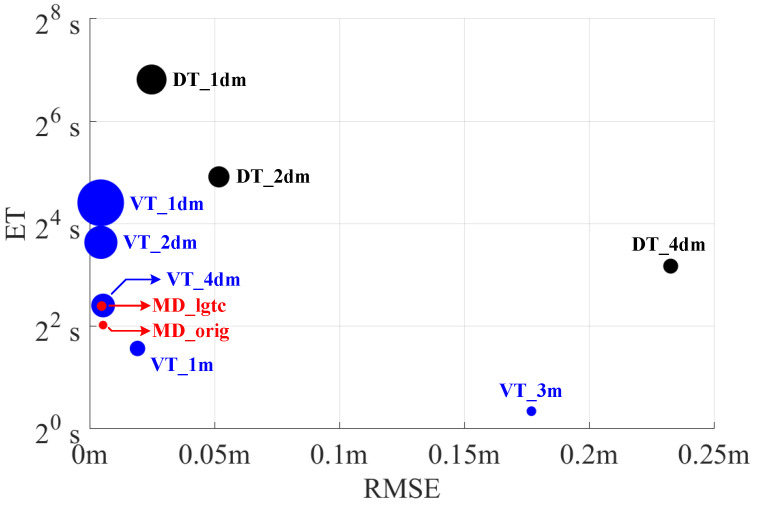
Illustration of performance of three methods. The ETs are plotted in binary logarithmic scale because of the huge differences in their values while various sampling resolutions are used. The radii of the circles indicate the center point numbers. The larger the radius, the more center points are.

In order to evaluate the performance accuracy of each method, the maximal deviation (maxDevi) to the benchmark and the root mean square error (RMSE) of all 36 lanes have also been calculated and shown in Table 3. The benchmark centerlines are extracted by DT with a resolution of 0.01m. Considering that centerlines extracted from different methods have different resolutions, the accuracy is evaluated after sampling each centerline with a fixed step of 0.005 m. For a more intuitive comparison, the performance of the three methods is also shown in Figure 11, although the range of ETs and RMSEs was limited for illustration purpose. All results are instead reported in Table 3.

Generally, MD_orig (without ligature compensation) is fast in execution and produces accurate centerlines, with a maximal deviation of 119.3 mm and an RMSE of 5.4 mm through all 36 lanes. Most importantly, it provides the most compact representation of centerlines with 3675 center points, compared to methods based on DT and VT which achieve comparable performance in terms of maximal deviation and RMSE (see results of DT with 0.02 m and VT with 0.4 m, respectively, in Table 3). By considering the VT method that calculates a comparable number of centerline points (VT with 4 m in Table 3), MD produces much more accurate results in terms of maximal deviation and RMSE. The accuracy of MD is further improved through ligature compensation, marked as MD_lgtc. The RMSE decreased by 10.8%, with an increase of around 1.2 s on ET and 891 center points in the ligatures. Despite the extra consumption on ET and storage, MD_lgtc is still fast and compact.

VT has excellent performance in terms of time efficiency, when the resolution is low. Starting from the original sparse border points, VT produces a poor centerline extraction with a huge maximal deviation from the benchmark centerline. Its accuracy improves with higher spatial densities. However, the number of center points grows with it. With the point resolution of 1 m, the number of center points is almost three times bigger than the output of MD_orig, and it increases linearly with the resolution.

DT operates the slowest, and its execution time surges with an increased resolution. By doubling the resolution from 0.2 m to 0.1 m, the execution time increases by almost four times. Although the performance of DT improves with a higher resolution, it is computationally prohibitive.

**Figure 12:**
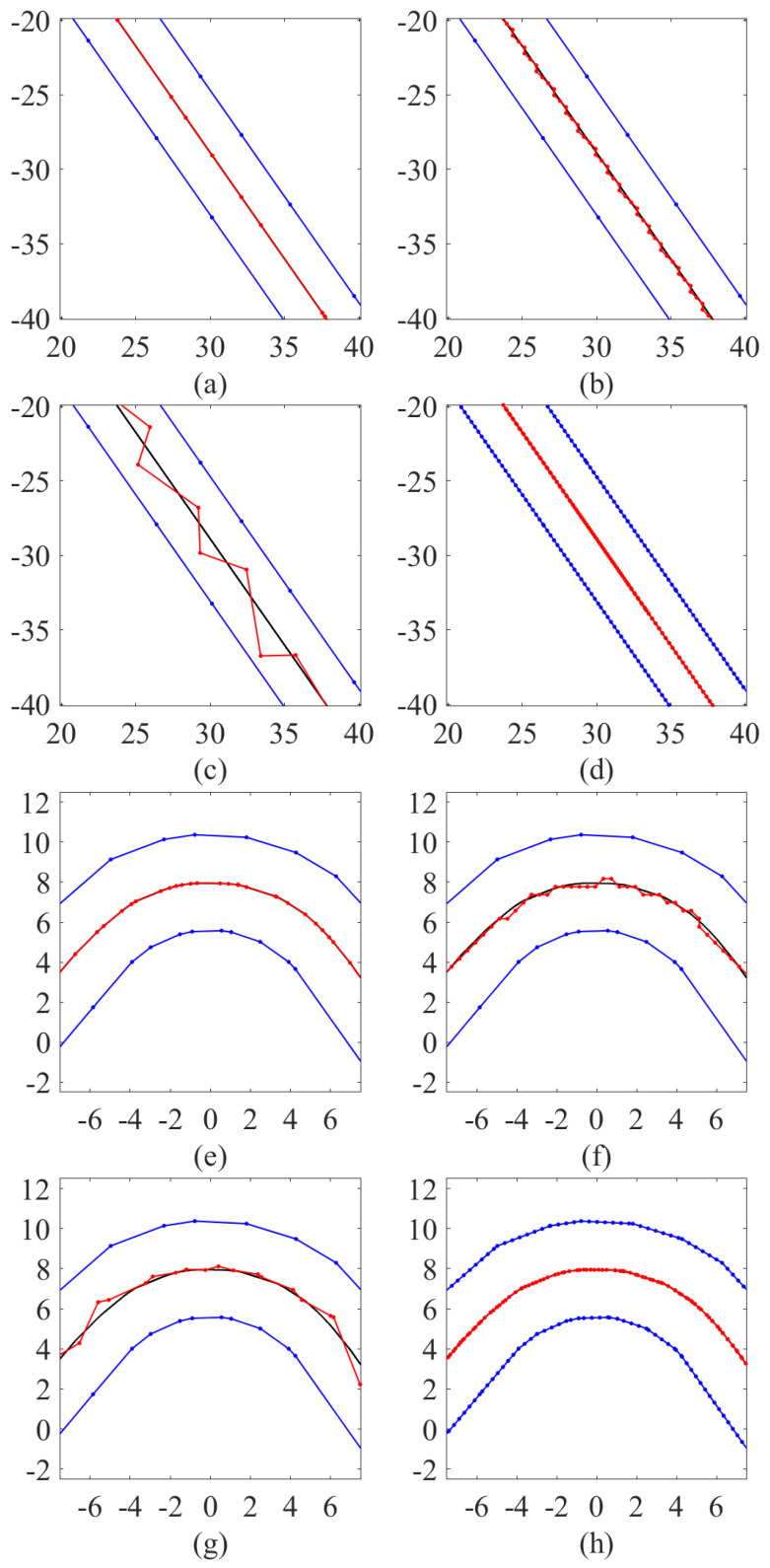
Illustration of the centerline extraction results of one lane. Lane boundaries are marked in blue while the ground truth and estimated centerlines are marked in black and red respectively. (**a**–**d**) show the results in the straight section and are obtained by MD_orig, DT (0.4 m), VT (original points) and VT (0.4 m) separately. (**e**–**h**) show the results in the corner section and are obtained by MD_orig, DT (0.4 m), VT (original points) and VT (1 m) separately.

**Figure 13:**
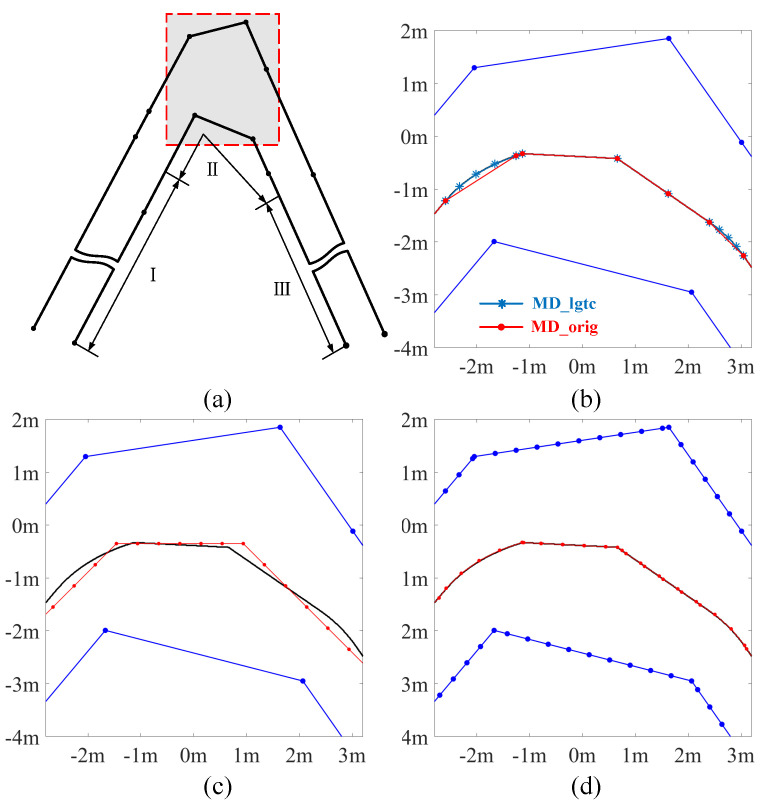
Illustration of centerline extraction performance in the sharp corner. (**a**) is the target lane and its centerline benchmark. (**b**–**d**) are results from MD, DT (0.4 m) and VT (0.4 m) separately.

To illustrate the performance of the 3 methods along the lane, the extracted centerlines are presented against their benchmark in Figure 12 and Figure 13.

MD and VT are evaluated on the original border points, to compare the performance of these two methods in sparse-point scenarios. Considering that DT only operates on a fixed resolution of the map, a grid of 0.4 m is selected to assess the performance of DT in low-resolution scenarios. Finally, the results of VT operating at a resolution of 0.4 m are also shown, to examine the performance of methods with similar accuracy to MD_orig. The results comparison is shown in Figure 12.

In both straight and bending sections, MD_orig shows a precise representation of the lane centerline. The accuracy of centerline extraction is high as the extracted centerlines are well-matched with the benchmark centerlines. DT provides a zigzag-like centerline in the straight lane and produces jags in the bending, which degrades the smoothness of the centerline. This is caused by the search in the discretized grip map.

The results from VT starting from the original sparse points are not smooth. It could be seen that where lane border points are not well aligned transversely, a protrusion occurs, which is very close to the border and may even cut across the lane polygon. In any case, there are visible deviations in the centerline, which are caused by the uneven distribution of lane border points. This problem can be mitigated by re-sampling the boundaries with a finer resolution, as shown by the performance of VT with borders sampled every 0.4 m.

A closer look at a sharp corner is shown in Figure 13. This is the 22nd lane in the self-created dataset and has a 75-degree corner. The max error of MD is always met in sharp corners like this one. From the picture, it is possible to see that the centerline extracted by MD_orig is flat compared to the benchmark. This is the ligature covered in Section 2.5. MD_orig performs an undercut in the sharp bending, underestimating the real centerline, because the method is not designed to track curved centerline. However, the start and end points of the ligature have been accurately calculated by MD_orig. It can be seen that MD_lgtc generates additional center points in the ligature, using more centerline segments to approximate the ligature curve and thus minimizing the deviation from the benchmark.

**Figure 14:**
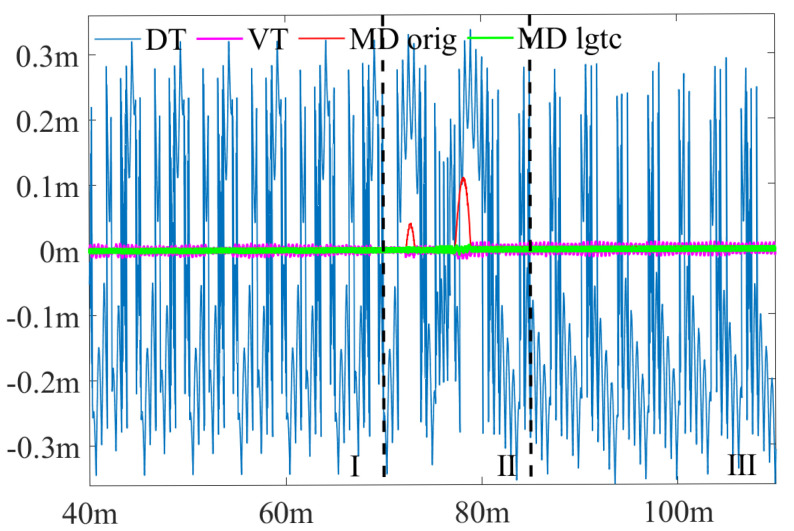
The deviation from the calculated centerline to the benchmark centerline. The green, red, pink and blue lines are from MD_lgtc, MD_orig, VT and DT separately.

To better illustrate the effect of the ligature and the effectiveness of MD_lgtc, the selected lane is partitioned into 3 sections, as Figure 13a shows, and the deviation along the benchmark centerline in these 3 sections is shown in Figure 14. The deviation of MD_orig in the sharp corner is obvious in the Section 2, reaching a maximum deviation of 0.1106 m. This deviation is minimized by ligature compensation in MD_lgtc. It could also be seen that, in the straight parts of the lane, MD_orig and MD_lgtc produce centerlines very close to the benchmark while VT and DT produce centerlines fluctuating around the benchmark.

As discussed, experiments were also carried out on the inD dataset to evaluate performances of the proposed method on real-world data of a complex intersection. In inD, some lanes, particularly non-driving ones, feature varying lane widths, sharp turning and irregular shapes. Examples have been given in Figure 9a,b, marked with red dashed lines. To better illustrate the performance of the MD in regular driving lanes and challenging non-driving lanes, results are evaluated separately, and can be found in Table 4 and Table 5. Generally, MD_lgtc consistently achieves better accuracy in terms of RMSEs while producing the fewest number of centrepoints. Compared to VT (0.4 m) which has a similar level of precision, MD_lgtc only generates about 30% points and features high storage efficiency.

Though VT (0.4 m) has a smaller max deviation compared to MD_lgtc, indicating its better ability to capture extreme deviations, MD_lgtc achieves smaller RMSE and thus higher overall accuracy across the complete lanes. The faded overall performance of VT is due to its sensitivity to the uneven distribution of boundary points. VT’s performance, particularly in straight sections, can be highly influenced by the spatial alignment of boundary points. If the boundary points are unevenly distributed, VT tends to produce larger errors. This is particularly evident in its performance as shown in Figure 14. In contrast, MD is less impacted by the alignment of boundary points, resulting in more stable performance across different lane configurations.

**Figure 15:**
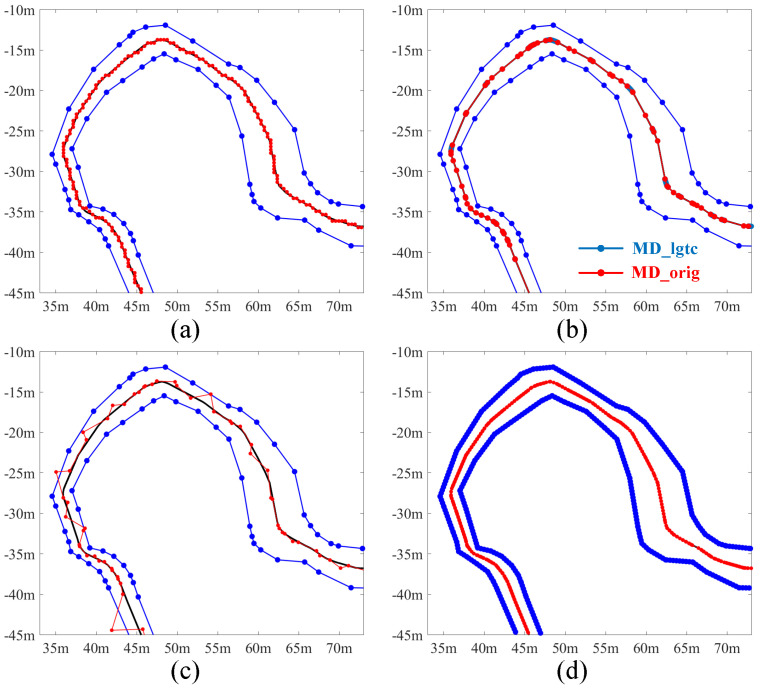
Illustration of centerline extraction performance in the lane with varying widths. (**a**–**d**) are results from DT (0.4 m), MD, VT (original points) and VT (0.4 m) separately. The lane boundaries are marked in blue and the ground truth centreline is in black, while the estimated centerline is in red.

To demonstrate the capability of the proposed MD-based method in accurately extracting the centerline despite variations in road width and the presence of bends, a non-driving path with these characteristics was selected from the map. This path is illustrated as red dashed lines in Figure 9b. The Figure 15b clearly shows that the MD-based method effectively extracts the centerline even when the road width changes continuously. In comparison, the centerlines extracted using DT (0.4 m) and VT (original points) appear zigzagged, neither smooth nor accurate, as shown in Figure 15a and Figure 15c respectively. Although VT (0.4 m) is also effective in extracting the centerline as shown in Figure 15d, it requires border intensification to enhance spatial resolution, which results in the introduction of more points and consequently increases storage requirements.

**Figure 16:**
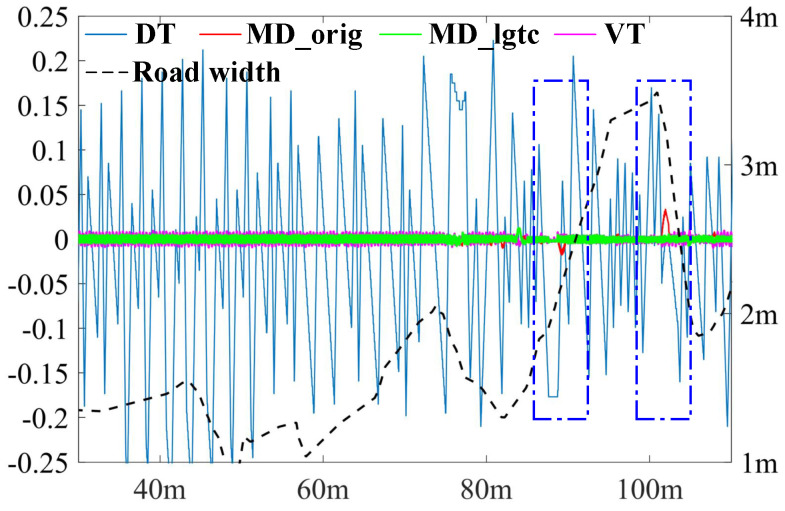
The deviation from the calculated centerline to the benchmark centerline. The green, red, pink and blue lines are from MD_lgtc, MD_orig, VT and DT separately. The black dashed line is the road width along the lane.

The impact of varying road widths on the accuracy of centerline extraction is more clearly illustrated in Figure 16. In sections where the road width changes sharply, as indicated by the blue dashed rectangle, the MD_orig method experiences a loss of accuracy due to the formation of ligatures. In contrast, the MD_lgtc method significantly enhances accuracy, thereby improving overall performance in these challenging segments.

## 4. Conclusions

This paper presents a lane centerline extraction method based on maximal disks (MDs), which is designed for optimal performance in sparsely defined driving scenarios.

The proposed method features light data storage and high execution efficiency. Results have shown that: (1) A smooth and accurate centerline could be obtained using this approach. (2) The proposed approach achieves higher accuracy compared to other methods like DT and VT in sparse point scenarios, or with small numbers of data points. (3) For the same target accuracy, the proposed method is significantly faster than the DT and VT. While the other two methods have been implemented with mature and efficient libraries, there is plenty of room for performance improvements in the implementation of the proposed approach.

Because of the minimal number of extracted centerline points, the proposed method can support efficient implementations of path planning algorithms. It can also be used to convert RL maps between different centerline-based or border-based formats, without increasing the number of data points.

Though ligatures have been identified and additional center points are generated to better approximate the curves in the ligature, there are some cases in which no MDs are identified due to constraints of abnormal lane shapes. This further leads to failure of ligature recognition and causes errors in centerline calculation. Future work will be focused on improving centerline extraction in these scenarios.

## Figures and Tables

**Table 1:** Statistics of lane boundary segments.

	numLanes	accuLgth [m]	avrgSegLgth [m]	maxSegLgth [m]	minSegLgth [m]
Sce1	27	2884	4.13	21.51	0.10
Sce2	34	4855	6.10	88.85	0.13
Sce3	20	1671	3.61	17.29	0.04
Sce4	22	4024	7.08	33.90	0.23
SCD	36	5473	5.31	36.19	0.35

**Table 2:** Statistics of road lane widths.

	avrgWidth [m]	maxWidth [m]	minWidth [m]	stdWidth [m]
Sce1	1.68	4.05	0.68	0.51
Sce2	1.64	3.44	0.59	0.50
Sce3	1.67	3.89	0.43	0.61
Sce4	1.71	4.48	0.75	0.51
SCD	2.08	3.65	0.45	1.02

**Table 3:** The comparison of results of 3 methods in the self-created dataset.

Method		ET [s]	Point Number	maxDevi of All Lanes [mm]	RMSE of All Lanes [mm]
MD	orig	4.053	3675	119.3	5.4
	lgtc	5.243	4566	119.3	4.8
DT	0.4 m	8.997	11,050	959.2	226.8
	0.2 m	30.109	22,136	207.9	51.7
	0.1 m	112.317	44,258	101.2	24.8
	0.02 m	2168.122	221,366	21.2	7.2
VT	sparse	0.692	2077	58,446.8	7458.7
	4 m	0.8205	3734	1664.1	319.9
	3 m	1.264	4598	1051.4	176.8
	1 m	2.953	11,718	161.3	19.2
	0.4 m	5.280	27,730	32.3	5.4
	0.2 m	12.405	54,290	15.6	4.5
	0.1 m	21.195	107,581	12.5	4.4

**Table 4:** The comparison of results of MD and VT in the inD: driving lanes (maxDevi and RMSE in mm).

Methods		Sce1		Sce2		Sce3		Sce4		Sce1–4
		maxDevi	RMSE	maxDevi	RMSE	maxDevi	RMSE	maxDevi	RMSE	Point Number
MD	lgtc	18.1	4.5	19.7	4.3	23.8	4.4	15.1	4.3	9782
VT	4 m	910.2	214.9	828.4	248.7	1236.8	236.6	812.9	222.1	4554
VT	0.4 m	15.6	5.1	16.4	5.0	16.6	5.3	13.6	4.8	33,734

**Table 5:** The comparison of results of MD and VT in the inD: non-driving lanes (maxDevi and RMSE in mm).

Methods		Sce1		Sce2		Sce3		Sce4		Sce1–4
		maxDevi	RMSE	maxDevi	RMSE	maxDevi	RMSE	maxDevi	RMSE	Point Number
MD	lgtc	93.9	5.9	88.1	5.3	36.8	4.5	35.8	4.3	10,777
VT	4 m	1173.5	241.5	1411.8	330.0	1970.5	450.8	992.7	313.2	4688
VT	0.4 m	17.4	5.5	20.1	5.7	24.5	6.3	15.5	5.2	33,728

## Data Availability

Data supporting this study are partially available from Results of Centerline Extraction Based on Maximal Disks at https://doi.org/10.57996/cran.ceres-2734 (accessed on 7 April 2025).

## Abbreviations

The following abbreviations are used in this manuscript:

accu	accumulated
avrg	average
DL	Deep Learning
DT	Distance Transform
DLT	Discrete Laplace Transform
ET	Execution Time
KDE	Kernel Density Estimation
lgth	length
lgtc	ligature compensation
LHS	Latin Hypercube Sampling
maxDevi	Maximal Deviation
num	number
MD	Maximal Disk
orig	original
RL	Road Layout
RMSE	Root Mean Squre Error
SAM	Steepest Ascent Method
seg	segment
SCD	Self-Created Dataset
Sce	Scenario
std	standard deviation
VT	Voronoi Tessellation
